# Exploring the Potential of Using Marine-Derived Ingredients: From the Extraction to Cutting-Edge Cosmetics

**DOI:** 10.3390/md21120620

**Published:** 2023-11-29

**Authors:** João Pedro Costa, Luísa Custódio, Catarina Pinto Reis

**Affiliations:** 1 Faculty of Pharmacy, Universidade de Lisboa, Av. Professor Gama Pinto, 1649-003 Lisboa, Portugal; joao.pedro.costa.712@gmail.com; 2 Centre of Marine Sciences, Faculty of Sciences and Technology, University of Algarve, Campus of Gambelas, Ed. 7, 8005-139 Faro, Portugal; 3 Research Institute for Medicines, iMed.ULisboa, Faculty of Pharmacy, Universidade de Lisboa, Av. Professor Gama Pinto, 1649-003 Lisboa, Portugal; 4 Instituto de Biofísica e Engenharia Biomédica (IBEB), Faculdade de Ciências, Universidade de Lisboa, Campo Grande, 1749-016 Lisboa, Portugal

**Keywords:** cosmetics, marine, extracts, marine products, blue biotechnology, sustainability

## Abstract

The growing understanding and knowledge of the potential of marine species, as well as the application of “blue biotechnology” have been motivating new innovative solutions in cosmetics. It is widely noted that that marine species are important sources of compounds with several biological activities that are yet to be discovered. This review explores various biological properties of marine-derived molecules and briefly outlines the main extraction methods. Alongside these, it is well known the legislative and normative framework of cosmetics is increasingly being developed. In this research segment, there is a growing concern with sustainability. In this sense, “blue biotechnology”, together with the use of invasive species or marine waste products to obtain new active ingredients, haven been emerging as innovative and sustainable solutions for the future’s cosmetics industry. This review also examines the regulatory framework and focus on the recent advancements in “blue biotechnology” and its relevance to the sustainable development of innovative cosmetics.

## 1. Introduction

The cosmetics industry is growing globally, and the discovery and use of new and innovative active principles is an important key for this sector [1]. The global market value for natural cosmetics is forecasted to reach $54.5 billion in 2027 [2], and there has been an increase of 5% in the discovery of new biologically active compounds per year [3]. Therefore, the marine environment can play an important role in cosmetics [3]. The oceans are host to significant biodiversity [1,4,5], with more than 250,000 species described living in a multitude of habitats [1], many of them producing active molecules that can be potentially used in new formulations [1]. Moreover, and, unlike other terrestrial sources like plants, marine flora and fauna can not only produce unique biomolecules in terms of chemical structures and biological properties, but can also be cultivated to produce a significant amount of biomass [6].

In this review, the different biological properties of marine-derived molecules are addressed. An approach to the challenges and implications concerning the extraction of these compounds, as well as a summary of the new and groundbreaking “blue biotechnology” breakthroughs and how they relate to a sustainable production of new and cutting-edge cosmetics, makes this review innovative, and differentiates it from other reviews that have already been presented in the past.

## 2. Methods

This overview uses a compilation of the online available literature on Google Scholar and Google platforms, as well as on PubMed database. The analyzed literature was from between 1990 and 2023.

The selection of literature was based on novelty, one of the main goals of this review, and variety, regarding year of publication and approached topics. The used keywords included “cosmetics”, “extracts”, “algae”, “biotechnology”, “sustainability” and “extraction”, alone or in combination.

Legislative and normative literature, especially European Regulations, were also used in this review.

## 3. Main Marine-Derived Cosmetic Ingredients

### 3.1. Skin-Whitening Agents

Skin-whitening products have been used across the world, with an especially significant market share in Asia [7]. When skin cells are irradiated by UV radiation, the damage of DNA is induced, with an increase in cAMP (cyclic adenosine monophosphate) and, consequently, MITF (melanocyte-inducing transcription factor), which results in the initiation of the transcription of pigmentation genes, including melanin [8]. Inhibiting the activity of tyrosinase, which catalyzes the rate-limiting step of skin pigmentation [7], is the most common among the different strategies to promote hypopigmentation [1,7,9]. Tyrosinase catalyzes, through a first reaction, the formation of DOPA (dihydroxyphenylalanine) and, in the second phase, the oxidation of DOPA to dopaquinone [2].

Hydroquinone is used to lighten the dark patches of skin, but its toxicity and adverse effects [2,10], such as contact dermatitis and exogenous ochronosis [10], together with a growing tendency towards sustainable and biological solutions, has motivated research on skin-whitening agents derived from marine products [4]. Azelaic acid, kojic acid and phomaligol A are some of the already marketed examples of compounds that are from natural sources [4,11].

Brown algae are sources of skin-whitening agents [2,10] which are believed to be safer than conventional skin whiteners [6]. Fucoidans (polysaccharides) and fucoxanthin (xanthophyll) possess tyrosinase inhibition activity, suppress tyrosinase-related protein 1 (TRP1) [10], and reduce pigmentation [12]. Furthermore, phloroglucinol and its derived polymers, phlorotannins [10], such as 7-phloroeckol [1,2], fucophloroethol [13], fucodiphloroethol [13], fucotriphloroethol [13] and dieckol [5,13], found exclusively in brown seaweeds [10,14], exhibit anti-tyrosinase activity [1,2,5,12]. Eckol and dieckol have reduced melanin synthesis in B16F10 cells (from a murine melanoma cell line) and dieckol was also reported as having an activity three times higher than kojic acid, with an IC_50_ of 2.16 µg/mL inhibiting mushroom tyrosinase [10].

SNA077, a crude extract of marine *Streptomyces* sp., has the potential to act as a potent and effective whitening agent, as it was shown to inhibit melanogenesis in the MNT-1 human melanoma cell line and in the mouse melanocyte cell lines Melan-a and B16 by downregulating melanogenic proteins in the cAMP/PKA (protein kinase A)/CREB (cAMP response element-binding protein) signaling pathway [15].

Meroterpenoids isolated from *Sargassum serratifolium* (Phaeophyceae), like sargahydroquinoic acid, sargachromenol and sargaquinoic acid, also decrease melanogenesis in melanoma cells activated by α-MSH (α-melanocyte-stimulating hormone), influencing CREB signaling pathways [16].

Another important, less common, strategy to promote hypopigmentation is through preventing the maturation or intracellular trafficking of tyrosinase, inhibiting melanosomal transfer. An example is niacinamide, a vitamin B3 derivative, which is found in fish in general [9].

### 3.2. Anti-Aging Activity

Skin aging is a complex process involving intrinsic and extrinsic mechanisms [2,17,18,19], resulting in macroscopical changes of the skin, such as xerosis, wrinkles, fine lines, laxity and vasculature prominences, and the appearance of benign neoplasms (e.g., seborrheic keratosis) [2,6,18,20].

The inevitable genetic and physiological changes that occur over time [2,13,17,18,19], with which ethnicity and hormonal and anatomical variations are involved [17], make up the intrinsic mechanism of skin aging, in which the production of progerin increases [2]. On the other hand, controllable and environmental variables, such as exposure to pollution, smoke, UV radiation and infections agents [17,19], are related to extrinsic skin aging [2,13,17,18,19], in which DNA alteration and damage occurs [2,18].

#### 3.2.1. Photoaging and Photo-Protective Activity

UV radiation, in particular UVA radiation because of its ability to penetrate the dermis [17], is estimated to account for about 80 to 90% of the skin aging process [17,18]. Globally, exposure to sunlight is responsible for a series of biochemical and physiological outcomes, such as the disruption of the extracellular matrix (ECM) turnover and of the dermal fiber network, DNA damage, formation of reactive oxygen species (ROS), increase in inflammatory mediators and activation of signaling pathways [2,17,18,19].

In photo-aged skin, the disruption of the ECM turnover process, characterized by an upregulation of MMPs (collagenases, gelatinases and stromelysins-1) and a downregulation of TIMPs (tissue inhibitors of matrix metalloproteinases) in keratinocytes and fibroblasts [18,21], results in the deterioration of collagen, elastin and other ECM components [17,18,19,22]. Together with an increased production of XPF (xeroderma pigmentosum factor) [18], wrinkles appear in the aged skin.

UV radiation also damages the genetic material through the dimerization of pyrimidine (UVB) and formation of ROS or free radicals (UVA) [17,18]. Mitochondrial, peroxisomal, membranal and cytosolic ROS are particularly important in the extrinsic skin aging process [19] since they influence numerous cellular processes, including the activation of the MAP-K p38/JNK/ERK/AP-1 signaling pathway that leads to the already-mentioned upregulation of MMPs [17,23] and, consequently, the increased degradation of the ECM [16]. Simultaneously, NF-kB and AP-1 play an important role in the balance between proliferation and apoptosis [17].

MAAs (mycosporine-like amino acids, such as porphyra-35, porphyra-334 or shirorine) and scytonemin are examples of seaweed-derived UV filters [6,13,16,22,24], which constitute alternatives to the other available UV filters, the use of which is controversial due to their environmental impact, sensitizing properties and potential endocrine disrupting effect [22]. MAAs absorb UV radiation in wavelengths ranging between 310 and 362 nm, offering a better protection efficiency when located outside the cell, whereas scytonemin has a maximum absorption at 386 nm [22].

Erebusinone, a tryptophan derivative isolated from the Antarctic sponge *Isodictya erinacea* with a similar chemical structure to 3-hydroxykynurenine, has possible photoprotective properties, absorbing UVA radiation with a peak absorbance of 370 nm [25].

Isolated from cod eggs (*Gadus morhua* L.), gadusol and gadusolate are chemical and mechanistically similar to mycosporines, making these compounds likely to be included in sunscreen formulations in the future [22,26]. However, studies still need to be carried out in order to determine the exact effectiveness of gadusol, especially what it comes to SPF (Sun Protection Factor) [22].

Marine bacteria, especially extremophiles, also produce photoprotective compounds. For instance, *Klebsiella aerogenes* produces extracellular semiconductor particles of cadmium sulfide (CdS) in response to environmental stress that absorb UV radiation [23].

#### 3.2.2. Inhibition of MMPs (Matrix Metalloproteinases)

As stated before, MMPs regulate the tissue remodeling process, the synthesis and secretion of cytokines and cell adhesion molecules and the degradation of components [2,27]. Suppressing and modulating MMPs’ activity in skin cells, either directly or via signaling pathways, is one of the strategies used nowadays in anti-aging formulations [2].

Phlorotannin (from *Eisenia bicyclis*, a brown seaweed) and phloroglucinol derivatives, such as eckol, dieckol, dioxinodehydroeckol and bieckol, are responsible for the inhibition of MMPs in human fibroblasts [7,13,16]. The extract of *E. stolonifera* seaweed, containing eckol and dieckol, showed an inhibitory effect of 76% and 66.7% on NF-κB and AP-1 gene reporter activity, respectively, which are genes that govern the transcription of MMP-1 [2,28].

Fucosterol, fucoidan and, to a lesser extent, fucoxanthin also present anti-MMP activity. Fucosterol, a phytosterol present in *Sargassum fusiforme* (formely *Hizikia fusiformis*, a brown seaweed), modulates AP-1 and TGF-β1 signaling, leading to a less expressive activation of MMP-1 [29]. In a similar manner, fucoidan, like other sulfated polysaccharides, not only inhibits the production of MMP-1 mRNA through the ERK pathway when fibroblasts are induced with UVB radiation, but also increases the expression of type I procollagen synthesis [13,16]. Also, through the inhibition of the ERK/JNK pathway, *Streptomyces* sp.-derived sarmentosamide is another promising anti-aging agent that reduces the expression of UVB-induced MMP-1 in normal human dermal fibroblasts [30]. Sargachromanol E, present in the extract of brown macroalgae *Sargassum horneri*, activates tissue inhibitors of metalloproteinase 1 (TIMP-1) and 2 (TIMP-2), inhibiting MMP expression more effectively than retinoic acid [2,16].

#### 3.2.3. Antioxidant Activity

Oxidative stress, which is related to ROS production, plays an important role in the skin aging process [2,6,19], especially in extrinsic aging, where UV radiation is the main factor [19]. ROS cause damage to cellular components, including proteins, DNA and membrane lipids [2,6], the latter related to lipid peroxidation [2], and activate numerous signaling pathways, such as MAPK/AP-1/NF-κB/TNF-α/IL-6-mediated inflammation-induced aging and p53/BAX/cleaved caspase-3/cytochrome c-mediated apoptosis-induced aging [31]. The production of ROS also activates the production of MMPs [31].

##### Carotenoids

Some of the most well-known antioxidants are carotenoids, such as β-carotene, astaxanthin, lycopene, torulene and torularhodin [1,32], all with an excellent scavenging activity.

Astaxanthin is widely distributed among crustaceans (shrimps, crawfish, crabs and lobsters) and microalgae, showing stronger antioxidant properties than β-carotene [13]. It scavenges ROS [1,13,32], inhibits lipid peroxidation [33] and the activation of the NF-jB transcription factor [13] and blocks the production of pro-inflammatory cytokines, COX-2 and nitric oxide [13,33]. According to different authors [16,23,34], algal extracts containing astaxanthin caused significant changes in SOD (superoxide dismutase) and GSH (glutathione), as well as having a protective effect on UVA-induced DNA damage in UV-A-irradiated human skin fibroblasts, human melanocytes and human intestinal Caco-2 cells. Extracts from a microalgae *Haematococcus lacustris* (formely *Haematococcus pluvialis,* a species of Chlorophyta from the family Haematococcaceae), the richest source of astaxanthin [8,22], show improvements in the skin’s macroscopic appearance regarding wrinkles and texture, demonstrating a relevant protection against photooxidative damage [13]. Isorenieratene, renieratene and renierapurpurin are other less well-known carotenoids isolated from marine sponges, including those from orders Poecilosclerida and Axinellida, that show similar oxygen scavenging and lipid peroxidation inhibitory activities to those of astaxanthin [6]. Another important pigment with established antioxidant activity, β-carotene, is also in use nowadays as provitamin A, especially that isolated from microalga *Dunaliella salina* (Clorophyta) [1].

##### MAAs and Other Pigments

Aside from the already-mentioned anti-MMPs and photo-absorbing activities, MAAs scavenge ROS, such as superoxide anion, and prevent lipid peroxidation [6]. Notably, mycosporine-glycine offers a fast protection against oxidative stress, even prior to the intervention of endogenous antioxidant enzymes [25]. The same goes for sargachromanol E; it already has a well-established anti-MMP activity, but research has also shown the suppression of UVA-induced intracellular formation of ROS and inhibition of lipid peroxidation [13].

Pigments, such as xanthophylls and chlorophylls, can also present antioxidant activity. Isolated from the acetone extract of the brown algae *Sargassum fusiforme*, fucoxanthin, a xanthophyll, also showed potent antioxidant activity against the DPPH free radical [13].

Cahyana et al. [35] detected a strong antioxidant activity of both acidic and neutral fractions of the extract of *Eisenia bicyclis*, an edible brown alga, through the ferric thiocyanate method, identifying a chlorophyll named pyropheophytin A as one of the contributors. This newly isolated compound showed a higher antioxidant activity than α-tocopherol [23].

##### Polysaccharides and Oligosaccharides

Regarding marine-derived polysaccharides, fucoidans, carrageenan and ulvans also present antioxidant properties. For example, superoxide and hydroxyl radical scavenging activity was observed in fucoidan isolated from *Saccharina japonica* (formely *Laminaria japonica),* while that from *Fucus vesiculosus* (Phaeophyceae) exhibited some ferric-reducing antioxidant power (FRAP) [36].

Isolated from red algae, carrageenan has an established use in cosmetic products as a stabilizer, emulsifier and moisturizer [37]. However, it also shows an interesting antioxidant activity, with κ-carrageenan exhibiting the highest DPPH-reducing capability and, consequently, the highest ROS-scavenging potential among the different carrageenan isomers, with a percentage removal of free radicals of 31.42% versus only 23.37% of vitamin E [36,37].

Extracted from the green algae *Ulva australis’* (formely *Ulva pertusa*) cell wall, sulfated heteropolysaccharides called ulvans have important biological properties, such as antioxidant activity and the ability to chelate ferrous ions. The higher the sulfate content in ulvans, the higher the antioxidant activity [36].

Lastly, agarose-derived oligosaccharides (AOSs), obtained from the chemical and enzymatic hydrolysis of agar, showed some antioxidative potency, especially regarding the radical scavenging capacity in DPPH assays. Agarohexose showed the best results, reducing 50% of oxidants generated by hydrogen peroxide at 1 mg/mL, but agarobiose, agarotetrose and agarohexaose also demonstrated antioxidant properties. In particular, agarohexaose can protect against ROS-associated *in vitro* cell damage [36].

#### 3.2.4. Anti-Inflammatory and Wound-Healing Ingredients

The exposure to sunlight leads not only to microvascular changes and the transendothelial migration of leukocytes [2], but also to the activation of proinflammatory genes, resulting in an inflammation cascade that triggers ROS production [2,13], which activates COX-2 and PGE2 [13]. In parallel, a transcription factor that regulated higher oxidative stress called NFjB is activated by ROS, which stimulates the expression of proinflammatory cytokines and COX-2 [2,13]. These molecules are produced in keratinocytes and are regulated by NF-κB, a transcription factor that regulates, among others, telomerase gene expression, inflammation and angiogenic activity [2].

##### Polysaccharides and Oligosaccharides

Different sulphated polysaccharides, including fucoidan, have shown in vivo anti-inflammatory activity in different studies [37]. Fucoidan stands out as the most frequently cited compound for these properties, making it our primary focus. Purified from *Fucus vesiculosus*, fucoidan from the brown algae *Turbinaria ornata* reduced the production of pro-inflammatory molecules, such as nitric oxide (NO), prostaglandin E2 (PGE2), IL-1β and TNF-α, and disrupts the MAPK and AKT signaling pathways in BV2 microglial cell line, as shown by the Western blotting applied [37]. Fucoidan from the brown algae *Turbinaria ornata* reduced the levels of inflammatory biochemical markers in cotton-pellet induced granulomas in rats, such as cathepsin D, myeloperoxidase (MPO) and C-reactive protein (CRP), somewhat comparable to dexamethasone [37]. Fucoidan can be presented in two main isoforms: low-molecular-weight fucoidan, hereinafter referred to as LMF, and high-molecular-weight fucoidan (HMF). LMF has a better bioavailability in tissues in dermal wounds created on the dorsal back of rats [5]. As stated by the study conducted by Park et al. [38], *Undaria pinnatafida*’s LMF is expected to act as a “wound-healing accelerator” [37,38] due to its anti-inflammation and angiogenesis activities [6]. LMF enhanced the wound-healing process, not only by reducing the recruitment of leukocytes, but also by promoting re-epithelization [38]. The use of fucoidan in topical cosmetic after-sun formulations [12] and in preventive and therapeutic agents against atopic dermatitis, through the inhibition of several chemokines [36], is also justified by the widely described wound-healing and anti-inflammatory properties.

##### Phlorotannins

Dieckol isolated from *Ecklonia cava* (a marine brown algae) suppressed, in a dose-dependent way and with no cytotoxicity, the production of nitric oxide (NO) and prostaglandin E2 (PGE2), but also other pro-inflammatory cytokines (IL-1β and TNF-α) [39]. The inhibition of NF-κB and p38-MAPK signaling pathways and, consequently, the inhibition of ROS production is another mechanism through which dieckol can prevent inflammation [39]. Other phloroglucinol derivatives, including phlorofucofuroeckol A and B, which downregulate iNOS and PGE2, inhibiting NO production, and diphlorethohydroxycarmalol, which also downregulates iNOS, COX-2 and NF-κβ, have also been described as anti-inflammatory compounds [2].

##### Coral-Derived Pseudopterosins

Corals are also a source of cosmetically interesting compounds. The most relevant ones are *Pseudopterogorgia elisabethae*-derived pseudopterosins A, B, C and D, a group of diterpene glycosides that possess various biological activities including anti-inflammatory, analgesic, antibacterial and anti-acne activities [6]. These glycosides are already being commercialized as Resilience^®^ by Estée Lauder [6]. Pseudopterosin A is the most-studied compound. Its anti-inflammatory properties differ from those described so far, as it inhibits phagosome formation and triggers the G-receptor-mediated release of intracellular calcium ions [6].

##### Sea Cucumber-Derived Fatty Acids

Sea cucumbers are known in folk medicine for the treatment of wound healing, being used to accelerate the wound contraction rate [6]. Some of the most common bioactive compounds in sea cucumbers include saponins, collagen, vitamins A, B1, B2 and B3, amino acids, bioactive peptides, minerals, fatty acids and gelatin [6].

*Stichopus hermanni* (Echinodermata)-based hydrogel has numerous advantages, mostly due to the immobilization of biological active compounds for a longer period in the matrix, which creates a controlled release system that easily interacts with the wounds and facilitates the healing process at a later stage. This hydrogel, which was created by incorporating sea cucumber in a standard hydrogel formulation, showed the enhanced histological reorganization and modulation of the inflammatory responses, with a significant reduction in pro-inflammatory cytokines (such as IL-1α, IL-1β and IL-6), in deep partial skin thickness burn wound in rats [40].

Globally, studies have been corroborating that aqueous extracts of sea cucumbers are much more cosmetically interesting than organic extracts because of their content in fatty acids and antioxidants, the latter playing an important role in controlling ROS production at wound sites [6]. For example, decosahexaenoic acid (DHA) from *Stichopus chloronotus* (Echinodermata) has been linked to the stimulation of pro-inflammatory cytokine production at wound sites, leading to a stimulation of the migration and proliferation of skin cells and to a breakdown of ECM proteins [6]. Moreover, the major fatty acids in sea cucumbers, both DHA and EPA (eicosapentaenoic acid), also stimulate the production of resolvins (inhibiting IL-1β) and protectins (inhibiting TNF-α and IL-1β) via COX-2 and 5-LOX (5-lipoxygenase) pathways [6]. Saponins present in the extracts of sea cucumbers have also been linked to the prevention of TNF-α production by NF-κB [6].

The improvement in the levels of TNF-α after the incorporation of sea cucumber extracts into Carbopol^®^ gel base in diabetic foot ulcer patients is a practical example of the benefits of these marine organisms in wound healing and inflammation [41].

#### 3.2.5. Collagen

Collagen, the main structural protein in the ECM, plays a structural role in supporting the formation, tensile strength and flexibility of joints [42]. The different types of collagen, namely types I, II, III, V and XI, organize themselves into fibrils that allow for support and resistance to mechanical stress in connective tissues [42].

One of the common sources of collagen is bovine and porcine skin. However, a series of bovine spongiform encephalopathy cases, as well as religious issues, limit its use [42,43]. Therefore, marine collagen arises as an important alternative, commonly isolated from fish, jellyfish and sponges [42]. Aside from accelerating wound healing through increased vascularization and epidermal growth and regenerating bone [42], marine collagen has been showing to possess anti-aging properties through the reduction in wrinkles and improvement in skin elasticity, structure and appearance [42]. Furthermore, marine-derived collagen has also shown ROS-scavenging activity, with antioxidant properties [44].

Collagen derivatives, namely marine collagen peptides (MCPs), have also shown advantages regarding skin and bone repair [44]. These MCPs are obtained via the enzymatic digestion of collagen [44,45], using trypsin for example [45]. MCPs are considered anti-aging compounds, since they promote photoprotection and immunomodulation, as well as the improvement in the premature senescence of the skin cells [44]. In particular, Pozzolini et al. [45] suggest that MCHs (marine collagen hydrolysates) derived from the marine sponge *Chondrosia reniformis* can be used both in drug and cosmetic formulations for damaged or photoaged skin repair due to its capacity to stimulate cell growth.

### 3.3. Anti-Acne Activity

Acne vulgaris is the most common skin disease, characterized by the chronic inflammation of the pilosebaceous unit [6,46]. It is a multifactorial disorder, in which hormonal, microbiological and immunological mechanisms can be taken into account, and exacerbated sebum production, the hyperkeratinization of the follicles and bacterial proliferation are some of the main factors that contribute to acne’s severity and progression [10,46].

Regarding bacterial proliferation, *Propionibacterium acnes* and *Staphylococcus epidermidis* are the main microbiological targets because they stimulate an inflammatory environment through the release of ROS and cytokines and the activation of TLR (Toll-like receptors) both in early-stage and late-stage acne inflammation [6,47]. *P. acnes* also releases lipases that digest the excess skin oil and sebum, resulting in local inflammation [6].

Sargafuran, derived from *Sargassum macrocarpum* (Phaeophyceae)*,* has antibacterial activity against *P. acnes*, with a minimum inhibitory concentration (MIC) of 15 µg/mL [6], which may be useful in new skincare cosmetics to prevent acne [2,48]. In addition, *E. bicyclis*-derived phlorotannins (a brown alga from the family *Lessoniaceae*) also demonstrate an effective inhibitory activity against *P. acnes*, *Staphylococcus aureus* and *S. epidermidis*. The latter is also inhibited by carrageenan, from red algae, with an MIC of 0.325 mg/mL, and by sulfated galactan, also from red algae [16].

Diterpenes, originating from soft corals, including cembrene diterpenoids, display several biological properties of interest to the cosmetic industry. Chen et al. [49] demonstrated that sinulariolides from *S. flexibilis*, such as SC-2, SC-7 and sinularin (SC-9), inhibit keratinocyte over-proliferation and anti-NO production properties. SC-9 also inhibited sebum secretion. *Sinularia flexibilis* is a species of soft coral in the family Alcyoniidae. Overall, these compounds show great potential to be integrated in anti-acne formulations.

Brominated compounds, isolated from Rhodophyta species (red algae), constitute a wide group of anti-acne molecules. Ranging from simple compounds like bromophenols or bromoform to more complex molecules such as organobromine compounds, these exhibit antibacterial activity against *P. acnes* and *S. epidermidis*. *Symphyocladia latiuscula*, a red algae (Rhodomelaceae) that is predominantly distributed along the coasts of Korea, Japan and northern China, contains high amounts of bromophenols, which are toxic to some bacteria, demonstrating the ability to inhibit *C. acnes* development, with an MIC of 0.21 mg/mL [46].

Osmundaria serrata’s lanosol ethyl ether, another brominated phenol, is highly bacteriostatic and mildly bactericidal, with an MIC of 0.08 mg/mL [46]. *Asparagopsis armata*’s organobromine compounds have also demonstrated the significant inhibition of a *P. acnes* culture [46].

### 3.4. Formulation Promoters and Facilitators

So far, we have been discussing the different uses of marine-derived products as cosmetic active ingredients. However, the marine environment also provides interesting excipients that facilitate and promote more cosmetically appealing formulations for consumers. Excipients are also indispensable for the product’s long-term stability, quality and microbial resistance to contamination [5].

#### 3.4.1. Sea Water

There are a very limited number of cosmetics, specifically powders, lipsticks and nail polishes, that do not include water in their formulations. Given that fresh water is a limited resource, sea water can be an interesting alternative, since it is a well-known source of minerals, including chlorides, magnesium, sodium, calcium, potassium, bromides, sulfates and bicarbonate, with benefits for inflammatory skin disorders, such as atopic dermatitis [22].

#### 3.4.2. Polysaccharides as Gel-Forming Agents and Viscosity Controllers

Polysaccharides and oligosaccharides include carrageenans, alginates, agar, laminarin, fucoidan, xylans and mannans. The cosmetic properties of different marine-derived polysaccharides and the species in which these compounds can be found are depicted in Table 1.

Alginates and their derived salts can be obtained from brown marine macroalgae, with their cosmetic properties being linked to physical properties, biocompatibility and biodegradability [22]. The application of alginates in microencapsulating materials for new cosmetic formulations, including those involving a controlled release, is also important, especially at a pH higher than 3-4, for better stability [5,22]. At a low pH, these polysaccharides also solidify and stabilize emulsions in a highly efficient manner [12].

Carrageenans are already widely used in cosmetics, such as creams, shampoos, sticks, sprays, and foams [5,12], with gel-forming, emulsifying, thickening and stabilizing properties, in addition to those described in Table 1 [5]. These can form single and double helices, which is responsible for their gelling capacity [5], depending on the concentration, temperature, presence of other solutes and the type of carrageenan used [22].

Agar, mainly formed by agarose and agaropectin, is found in the cell wall of red macroalgae and acts as a gelling, emulsifying and suspending agent [5]. The use of agar as an excipient has been suggested in creams, lotions, deodorants and anti-aging and anti-acne formulations [12].

Agarose, a natural polymer of galactose also extracted from red seaweed, is a candidate for an emulsifier and thickener used in cosmetics. However, due to its strong hydrophilic and gel properties, algae-derived agarose needs to be chemically modified in order to gain hydrophobicity, reducing its gel strength and giving it amphiphilicity [50].

Finally, fucoidan, extracted from brown macroalgae, can be used as a drug carrier for controlled release systems in skin formulations, as a wall component of several pharmaceutical forms, such as microparticles, nanoparticles, hydrogels and nanocapsules [5].

#### 3.4.3. Carotenoids, Chlorophylls and Phycobilins as Colorant and Dyes

The use of artificial colorants dates back to 1850, and they generally have azo dyes/heavy metal in high concentrations, which are harmful to the environment and positively correlated with several types of cancer. Consequently, natural colorants and dyes benefit from a better reputation and acceptance by consumers [51]. Macroalgae and cyanobacteria are sources of colorant molecules, such as carotenoids, chlorophylls and phycobilins, with a lower side effects profile and a variety of colors—blue, yellow, orange and red [5,22]. As an example of carotenoid, the natural product astaxanthin, derived from microalgae, represents the highest quality astaxanthin with the highest antioxidant potential. The global production of natural astaxanthin has steadily increased by an average of 11.23% per year [52]. Another example of a carotenoid is fucoxanthin, which is a pigment mainly associated with the chloroplasts of brown micro- and macroalgae, which represent approximately 10% of the total carotenoids in nature. In response to the increased interest of this carotenoid, the annual growth rate of the current market of fucoxanthin as a nutraceutical or cosmetic ingredient is 2.47%, on average [53].

Phycobillins, chemically tetrapyrroles, constitute the main photosynthetic accessory pigments from some algae of Glaucophyta, Crystophyceae and Rhodophyta groups, and are covalently bound to proteins, forming phycobiliproteins [22].

Flavonoids are a highly diverse group of plant secondary metabolites derived from the phenylpropanoid metabolism. Most flavonoids have been isolated from seagrasses (51%) and halophytes (28%) [54]. They can be divided into six major categories: flavanones, flavones, isoflavones, flavonols, catechins and anthocyanins. Specifically, most flavanones, flavones, isoflavones and flavonols are light yellow or yellow-colored, whereas anthocyanin hues range from red, pink and magenta to violet, purple and blue. Their health-promoting bioactivities, together with their diverse colors and high solubility in water, broaden their usage in the field of cosmetics [55].

However, the potential use of these compounds comes with some formulation challenges. Chlorophylls and carotenoids are lipophilic molecules, requiring organic solvents for their extraction (e.g., methanol or DMS), which should be avoided in cosmetics due to their toxicity [5]. On the other hand, water can be used in extracting phycobiliproteins, as they are polar molecules [4], but it will ultimately alter the organoleptic properties of some formulations, as water should not be used in make-up [22].

#### 3.4.4. Marine Biosurfactants

Marine biosurfactants are byproducts of marine microorganisms’ metabolism. In bacteria, for example, these molecules are produced so that they can use substrates that are not water-soluble [37]. These amphiphilic molecules, containing both hydrophilic and hydrophobic domains, allow hydrophobic substances to be more easily solubilized in water by reducing the interfacial tension. Biosurfactants have low critical micelle concentrations (CMC), which allows lower concentrations of these compounds to be used when compared to chemically produced surfactants [37,55].

Biosurfactants can be classified as high-molecular-weight (HMW) biosurfactants or bioemulsifiers and low-molecular-weight (LMW) biosurfactants, the latter including fatty acids and lipoaminoacids [55]. Sophorolipids (e.g., in *Paracoccus*) and rhamnolipids (e.g., in *Pseudomonas aeruginosa*), especially, have emulsifying and solubilizing properties, among others, justifying their potential use in cosmetic formulations [14]. Advantages in the use of marine biosurfactants include lower irritancy to the skin when compared to their synthetic counterparts [14] and their low eco-toxicity, meaning they have an acceptable environmental impact [23].

#### 3.4.5. Preservatives

Preservatives are crucial in this process of ensuring microbiologically free and stable cosmetics. Synthetic preservatives, including parabens, benzyl alcohol and EDTA (ethylenediaminetetraacetic acid), are known to cause allergic reactions. Natural preservatives, in which marine-derived compounds are included, are thus an alternative [2].

Polyphenols like flavonoids, found in several species of algae, have significant antibacterial activity [22].

*Gracilaria dendroides* (red macroalgae), for example, contains high concentrations of rutin, quercetin and kaempferol (10.5 mg/kg, 7.5 mg/kg and 15.2 mg/kg, respectively), which are able to successfully inhibit *E. coli*, *P. aeruginosa*, *S. aureus* and *E. faecalis*. These compounds were extracted using ethanol, chloroform, petroleum ether and water, and the antibacterial activity of each compound was determined using the agar diffusion technique, with ampicillin, oxacillin and ceftazidime as controls [22].

Laurinterol from *Laurenicia pacifica* (Rhodophyta) has antibacterial properties against *Staphylococcus aureus* [2]. Crude organic extracts from *Ulva lactuca* demonstrated an antimicrobial activity comparable to BHA or BHT, with minimal inhibitory concentration values, ranging from 0.40 to 0.35 mg/mL, especially against Gram-positive and Gram-negative bacteria [5].

## 4. Extraction of Marine-Derived Cosmetic Ingredients: Challenges and Implications

Globally, extracts can be obtained through several unit operations, including upstream processing (preparation for cultivation), cultivation in photobioreactors and downstream processing, in which cell harvesting, rehydration, extraction and ultrafiltration are included [56].

Conventional methods of extraction include infusion, percolation, Soxhlet extraction, maceration and steam distillation, among others. Advanced, alternative or contemporary extraction methods include supercritical fluid extraction (SFE), microwave-assisted extraction (MAE), ultrasound-assisted extraction (UAE), enzyme-assisted extraction (EAE) and electro-technologies. A short overview of the existing methods of extraction [2,54,56,57,58,59,60,61,62,63] can be consulted in the Supplementary Materials.

### 4.1. Challenges Regarding the Extraction and Utilization of Cosmetic Ingredients

Despite all the benefits and potentialities that come with the utilization of marine-derived ingredients in cosmetics, it is important to consider the different biological, technical and supply challenges, as well as problems regarding contamination and reproducibility of extracts obtained from raw materials.

#### 4.1.1. Biological and Technical Challenges

It is undeniable that marine biodiversity is both an advantage and a disadvantage for the cosmetic industry and manufacturers—on the one hand, there is a considerable number of compounds that can be explored in cosmetic formulations, but identifying and exploring this diversity in a sustainable and safe manner is still challenging [64].

Obtaining marine raw materials remains very difficult, and robotic and engineering advances are still relied on regarding the access to the ocean, especially the deepest locations. Thus, most studies are conducted in shallow coastal waters due to difficulties in reaching deeper spots (normally more than 30 m), leading to gaps in biodiversity and chemo-diversity knowledge. ROVs (remotely operated vehicles) are an interesting solution to this problem, but are very expensive and unattractive in developing countries from the tropical and subtropical regions, where these natural products are most likely unknown [64].

Access to biodiverse natural resources is now included in the Convention of Biological Diversity (CBD), in which “the potential role of access and benefit-sharing to contribute to the conservation and sustainable use of biological diversity” is acknowledged [65]. The use of marine products for biological purposes implies a correct taxonomic identification and classification; an incorrect taxonomic identification and classification can, however, compromise the entire cosmetic discovery project and lead to problems in the reproducibility of the extracts obtained. This is still considered a challenge for the use of marine-derived products, both due to the lack of taxonomic knowledge and due to the large number of undiscovered and undescribed species [64].

#### 4.1.2. Sustainable Supply

Like their terrestrial counterparts, marine organisms have a high biological and biochemical variability, particularly when considering the quantity and variety of metabolites generated based on the specific environmental conditions in which they grown [64]. Ensuring a sustainable source can be quite demanding when it comes to harnessing natural products, either because the desired compound exists in minimal quantities within the raw material, which can be influenced by external factors, or because isolating these compounds poses significant challenges. Hence, large quantities of raw material are necessary [57]. Efforts have been made to address the issue of sustainable supply, such as the optimization of molecular biology and aquaculture technologies, the latter including, for example, IMTA (Integrated Multi-Trophic Aquaculture) and RAS (Recirculating Aquaculture Systems), yet it remains a persistent and significant challenge [64,66].

Therefore, the development of synthetic or hemisynthetic analogues has been applied. However, due to the intricate nature of naturally derived molecules, which often feature multiple stereocenters, and the complexities involved in the purification processes, executing this approach becomes intricate and challenging. The need for the virtual screening of molecules and the misassignment of natural products are also key points [64].

#### 4.1.3. Reproducibility of Extracts and Challenges in the Scale-Up Process

The reproducibility of extracts and the expansion of cultivations from a laboratory scale to an industrial unit are some of the most significant challenges industries face when dealing with natural products in general.

Conventional extraction methods show low reproducibility, because of the lack of automation. Therefore, using advanced methods of extraction, such as supercritical fluid extraction (SFE) or electro-technologies, can be beneficial, both due to better reproducibility between extract batches and due to sustainability [56].

As previously mentioned, natural products alter their metabolite levels and growth rates in response to environmental conditions as a protective mechanism [66]. Different factors, including temperature, light availability, oxygen levels, mixing parameters, nutrient levels, risk of contamination or infestation, biomass film formation, and loss of the bioreactor’s transparency are some well-known key factors that influence macro- and microalgal growth [67,68]. For example, the protein levels of the red macroalgae *Palmaria palmata*, harvested in the French Atlantic coast, were found to fluctuate annually, ranging from 9% to 25%, with a peak in May [54]. Strategies to harmonize metabolites’ production in algae has been used due to the increasing research on this topic. For example, red light photons have been shown to accelerate the cell cycle, and green light has been shown to increase lipid production [67].

The scale-up process is crucial in process development [68], and is usually linked to significant productivity losses since optimal conditions determined in a laboratory scale are drastically different from those at an industrial scale [67,68]. The real-time monitorization of the temperature and pH of each photobioreactor, in the specific case of microalgae, is an important approach to standardizing and optimizing the quality of the produced biomass. The management of the cooling system and harvest during the semi-continuous phase on a daily basis are also strategies to overcome set challenges, allowing for different growth conditions, whether optimal or to increase the production of certain metabolites, and higher consistency between batches [67,68]. Before the full implementation of the commercial plant, data obtained from the scaling-up stage must be subjected to rigorous analysis and consideration, and the economic aspects of the process should be considered [68].

#### 4.1.4. Potential Contamination of Raw Material and Extracts

The contamination of raw materials and, consequently, of the obtained extracts is a significant concern when incorporating natural ingredients into cosmetic formulation and production. We will delve into two specific types of contamination, namely biological and chemical, in the following sections.

##### Biological Contamination

Biological contamination can be divided into two groups, namely a cell-growth-affecting contamination and a protein-accumulation-affecting contamination. The latter is related to the contaminant’s ability to reduce or consume the molecules produced by the organism of interest. Sources of biological contamination include, for example, aquatic and air pollution and the accumulation of nutrients, salt and other organisms in the blind angles of bioreactors used for microalgae cultivation [69].

Blind angles are common in photobioreactors with different materials and structures; in these spots, nutrients, microalgae and cyanobacteria accumulate, accelerating the corrosion of the equipment and the contamination of the next cultivation [69].

In order to reduce these problems, the optimization and control of growth conditions and the application of standardized procedures can ensure a constant and repeatable level of contaminant-free, microbiologically pure components in each raw product unit [58]. Special attention should be paid to heterotrophic cultures of microalgae, as bacteria, fungi and yeasts can ruin an entire cycle of production due to their high rates of growth and productivity [68].

The choice of media is also fundamental for this purpose. An artificial medium can, for instance, substitute supplemented municipal domestic wastewater in a culture of algae, reducing the probability of water-related contamination. When wastewater is effectively used in the growth media, pH control can prevent grazers’ contamination (such as zooplankton). The use of detergents or phenols can also be an alternative for bacterial contamination [68].

##### Chemical Contamination

Arsenic (As), cadmium (Cd), chromium (Cr), copper (Cu), lead (Pb), nickel (Ni) and zinc (Zn) are the most commonly found heavy metals in the water environment, and have significant environmental and evolutionary toxicity [22]. Algae, especially macroalgae, absorb heavy metals from marine water, through a bioaccumulation process [22,70]. Chang et al. [70] demonstrated the presence of arsenic (As, 3.9 ppm), iron (Fe, 14.9 ppm) and zinc (Zn, 3.0 ppm) in *Eucheuma cottonii* (a red macroalgae) collected in a coastal area of Malaysia. In a study developed with seaweeds from the Venice lagoon, a group of investigators found high contamination levels of lead in *Ulva* sp. and, to a lesser extent, in *Gracilaria* sp., and *Cystoseira* sp. was highly contaminated with arsenic [71].

In addition to heavy metals, iodine (I) is also present in ocean-bound algae and other marine products [56]. However, both heavy metals and iodine are prohibited in cosmetics according to the EU regulation on cosmetic products [72]. Note should be taken of Article 17 of the European Union regulation on cosmetic products; it states that “the non-intended presence of a small quantity of a prohibited substance, stemming from impurities of natural or synthetic ingredients, the manufacturing process, storage, migration from packaging, which is technically unavoidable in good manufacturing practice, shall be permitted provided that such presence is in conformity with Article 3” [72].

Although the presence of heavy metals in marine products is permitted, there is still a lack of information regarding their presence in cosmetic products. However, these chemical elements are expected to be found in their extracts, which will later be incorporated in cosmetic products. In a study by Grillo et al. [73], the heavy metal content in the extracts of two different species collected in the Venice lagoon was determined prior to incorporation in a cosmetic formulation—*Sargassum muticum* (Heterokontophyta brown algae) and *Ulva lactuca* (green algae). These extracts were obtained through MAE (microwave-assisted extraction) in a hydroalcoholic solution (70% EtOH/30% water). Arsenic (As) was not found in any samples, but nickel (Ni), chromium (Cr), lead (Pb), cadmium (Cd) and cobalt (Co) were found in the algal extracts. Particularly in *S. muticum*, the levels of nickel and chromium exceeded the legal limits imposed by the already-mentioned Article 17 [73]. In order to reduce the contamination of the final product in levels exceeding the imposed legal limits, the authors suggest a different harvesting location for *S. muticum* [73].

Periodically, and due to oil spills, for example, the contamination of seawater by VOCs (volatile organic compounds), such as various alkanes and benzene compounds, is also possible, and they have already been found in *Ericaria corniculata* (formerly *Cystoseira corniculata*, Phaeophyceae) and *Jania rubens* (Rhodophyta) [22].

### 4.2. Public Policy and Regulatory Framework

The use of marine-derived products in cosmetic products still needs further development regarding its regulatory framework, even though its use is increasingly common in the cosmetic industry.

CosIng, EU’s cosmetic ingredient database [74], along with Regulation 1223/2009/EC [71], contain all legal requirements and restrictions on each substance. There are several permitted marine-derived cosmetic ingredients in this database, such as fucoidan and carrageenan [74,75]. In the EU, a clear science-based regulatory environment is established, in which cosmetic claims need to be supported by adequate scientific evidence and address certain determined criteria [75]. ISO standard guidelines, such as ISO 16128-1:2016 [76], regarding natural and organic cosmetic ingredients, and ISO 16128-2:2017 [77], regarding the quality criteria for ingredients and products, are also important tools for the cosmetic industry, even though they do not have legal and regulatory power [75].

In addition to the EU’s regulatory documents and ISO guidelines, CEN, the European Committee for Standardization, provided two technical reports (TR) about the use of algae in cosmetics—CEN/TR 454 [78] and CEN/TR 17611 [79]. The latter will be further discussed below. CEN/TR 454 (standards for algae) states that algae-derived raw materials should be treated in the same way as plant-derived materials, addressing possible quality and contamination issues with specific standards [78].

#### CEN/TR 17611 Algae and Algae Products—Specifications for Cosmetic Sector Applications

This technical report was formulated in January 2021 in response to a request from the European Commission for CEN to create a standardized guideline on this subject. In this technical report, several important aspects are mentioned regarding product characteristics, product information documents, traceability, sustainable development and labelling [79].

Regarding purity, for example, the presence of GMO (Genetically Modified Organism) material and non-organic material in algae and algae products is considered as impurity, and can be determined with macroscopical/microscopical characterization and other identification tests. All powdered materials should be analyzed through microscopical characterization [79].

Heavy metal, physical (e.g., plastic fragments) and microbiological forms of contamination are some of the subjects addressed in this technical report. The consideration of long-term safety aspects is stated to be needed, especially regarding local toxicity on skin and eye irritation and sensitization. Dioxins, PAHs (polycyclic aromatic hydrocarbons) and other xenobiotics are also some of the discussed contaminants [79].

## 5. Sustainability and Marine Biotechnology in the Cosmetic Industry

### 5.1. Ensuring Sustainability in Marine-Derived Cosmetics: A Life-Cycle Approach

Approximately 7 billion tonnes of the 9.2 billion tonnes of plastic manufactured between 1950 and 2017 were transformed into plastic waste, eventually ending up in landfills or being discarded, with the potential to disrupt habitats and jeopardize ecosystems [80]. While sustainability is currently gaining popularity as a trendy topic, it remains crucial for the well-being of our planet’s environment to address this issue. This is due to the significant ecological, economic and social impact that the cosmetic industry has, whether it pertains to packaging, production methods, or implications for the population. The demand for natural marine products in skincare is substantial, and it is imperative to guarantee their sustainable and eco-friendly utilization [64].

#### Fundamentals of Sustainability and Its Application in the Cosmetic Industry

According to the UN’s 1987 Brundtland Report on Environment and Development, sustainability involves a balanced consideration of three dimensions: social, economic, and environmental [81,82,83,84]. Sustainable development is thus defined as “development that meets the needs of the present without compromising the ability of future generations to meet their own needs.” [84]

Innovation and technology are considered important drivers of sustainability, and can concern products or processes [82,85]. The rise of “eco-friendly” cosmetics and a particular concern with the use of clean technologies and the rational use of natural resources has led to the use of life-cycle approaches (LCA) [82,86]. An LCA comprises three steps [81]: the definition of goal and scoping and processing of system boundaries; the construction of a life-cycle inventory, with inputs and outputs of relevance throughout the entire product’s life cycle; and the impact assessment of each life cycle phase, including areas for improvement.

LCA covers the product’s full life-cycle process in a holistic way and a way in which the different stages are interdependent, starting with the extraction of raw materials and including the production, formulation and recycling steps [82,87].

It is important to take into consideration that there are no materials that can be considered 100% sustainable; the environmental impact of using certain materials occurs throughout the whole cosmetics supply chain [88].

### 5.2. “Blue Biotechnology” and Sustainability

#### 5.2.1. Biotechnology, Marine Biotechnology and “Blue Biotechnology”

OECD defines biotechnology as the “application of science and technology to living organisms, as well as parts, products and models thereof, to alter living or non-living materials for the production of knowledge, goods and services.” On the other hand, marine biotechnology is defined as “[encompassing] efforts that involve marine bio-resources, as either the source or the target of biotechnology applications” [89]. Biotechnology can be considered an “umbrella term” [89], as it includes other sectors, such as “red biotechnology” (or medical, health and pharmaceutical biotechnology), “green biotechnology” (related to agriculture), “yellow biotechnology” (or environmental biotechnology) and “white biotechnology” (related to industry) [89].

**Figure 1:**
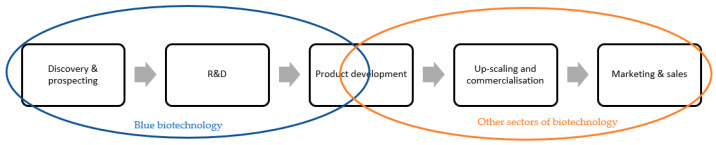
“Blue biotechnology” sector as a part of the product development pipeline. Discovery and bioprospecting involve the investigation of environments and collecting living organisms. In the R&D phase, active molecules are characterized and synthetic strategies are discussed. Finally, in the product development stage, a LCA is adopted and sustainable production strategies are developed. Adapted from Collins et al., 2018 [89].

“Blue biotechnology” is related to the use of marine resources as source materials, which is the only biotechnology sector to do so. Therefore, “blue biotechnology” is only applied to the first part of the development pipeline, including sampling, discovery and bioprospecting, research and development (R&D) and initial product development phases [89]. As represented in Figure 1, “blue biotechnology” ends in the early stages of product development; the subsequent stages are then inserted in the other sectors of biotechnology [89].

#### 5.2.2. Biorefineries in the Sustainability Assessment of “Blue Biotechnology” Processes

The EU Green Deal, which aims for climate neutrality by 2050, together with the consumers’ preferences with eco-cosmetics, have produced an important environmental awareness in the cosmetics industry [86]. Using a life-cycle approach and considering that “blue biotechnology” is only applied to the initial steps of a cosmetic’s life cycle, it is important to assess the different biotechnological processes regarding their sustainability.

Pagels et al. [86] conducted an environmental assessment of sea-harvested *Fucus vesiculosus* processing and compared it with the antioxidant profiles of vitamin C and green tea extracts, employing a LCA. Their study demonstrated that utilizing seaweed harvested from the sea entails significantly less human intervention and a notably higher growth rate, all without the need for terrestrial fertilizers, compared to land-based plants. Furthermore, seaweeds have a similar environmental load when compared to ascorbic acid, even presenting an advantage regarding marine eutrophication and water consumption [68,86].

**Figure 2:**
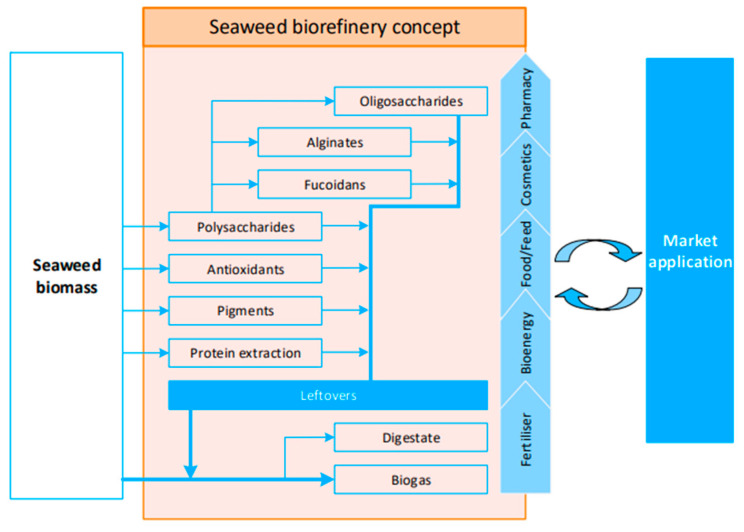
Seaweed biorefinery concept according to Balina et al., 2018 [90]. In this model, the production of biogas is included. It is important to note that the waste and leftover products obtained after each step of treatment are used as raw material inflows for a parallel production.

To reduce seaweed waste and maximize its diverse applications, the concept of a biorefinery can be applied. A biorefinery involves integrating various biomass conversion processes to generate both energy and value-added products, making it possible to establish such a facility. These processes align with a cascade transformation approach that is nearly zero-waste, simultaneously enhancing the efficiency of biomass conversion [90]. Figure 2 contains a seaweed biorefinery presented by Balina et al. [90].

A seaweed biorefinery concept can be used mainly to address the use of seaweed species whose overgrowth is causing ecological damage and disruptions to coastal environments due to eutrophication [90]. Pagels et al. [86] also introduced a biorefinery model suitable for cosmetics, where an initial extract is created for cosmetic applications, and any residual material is employed either as a fertilizer (acting as a biostimulant and biopesticide) or in the generation of biogas.

In addition to antioxidants, the production of astaxanthin from *Haematococcus lacustris* can also have remarkable benefits that are economic, social and environmental. As demonstrated by Pérez-López et al. [91], most of the environmental impact was associated with the cultivation stage, especially due to the production of electricity for artificial illumination and air supply. When sunlight was used instead of artificial illumination, this environmental impact was significantly reduced, but with a decrease in biomass productivity [90]. This process was economically favorable, with high profitability and low payback time, even considering less favorable conditions and a considerable level of uncertainty [91].

#### 5.2.3. “Blue Biotechnology”-Based Applications in the Cosmetic Industry

##### Marine Viruses as a Source of Ceramides

Astoundingly, marine viruses outnumber any living organism in the sea and have enormous potential in biotechnology, including in the cosmetic industry [92].

English researchers from the Plymouth Marine Laboratory and the Sanger Institute in Cambridge discovered that EhV-86, a marine virus, contains a cluster of at least seven genes that encodes several components of sphingolipid biosynthesis after the virus’ genome had been sequenced. These components then lead to the formation of ceramides, which have been increasingly used in the cosmetics industry as active ingredients due to their skin protection and hydration properties [92]. The use of ceramides from marine sources has several advantages, because, similarly to collagen, the animal sources used nowadays have a risk of contamination with pathogenic agents such as bacteria or prions (BSE, for example) [92].

##### Marine-Derived Biosurfactants

Surfactants and active surface agents are one of the fundamental components of a cosmetic formulation due to their cleansing, emulsification, foaming, solubilization and conditioning properties [37]. While this paper has previously discussed marine biosurfactants, we will now explore various sustainability-related advantages associated with their usage, along with providing some illustrative examples.

Artificial surfactants like sodium lauryl sulfate (SLS) and sodium laureth sulfate (SLES) are produced from petroleum-derived sources, leading to concerns about their impact on bioaccumulation, biodegradability and biocompatibility. These concerns extend to both the environmental and human health, with a particular emphasis on the latter due to multiple studies indicating that SLS and SLES can induce skin damage and irritation [37]. A biosurfactant obtained from *Nocardiospsis* VITSISB (a marine actinobacteria) allowed toothpaste to be formulated with a more optimal pH than the one formulated with SLS. Liposan and Yansan, biosurfactants from *Yarrowia lipolytica*, are also other examples. Yansan, specifically, has a higher emulsification activity, and its stability ranges from pH 3.0 and 9.0 [37].

##### Immobilized Lipases from Antarctic Fungi and Yeasts

Lipases are enzymes that naturally catalyze the hydrolysis of carboxylic ester bonds in hydrophobic compounds, exhibiting compatibility with fatty, non-aqueous media and emulsions found in cosmetics, as well as possessing a wide range of substrate acceptance [93]. In cosmetic products, lipases can be used both as active ingredients or as biocatalysts in the synthesis of specific cosmetic ingredients [93]. Examples of lipase applications as active ingredients include facial cleansing, anti-cellulitis treatments and body-slimming products. These molecules are responsible for mild skin peeling, as they affect the stratum corneum’s keratinocytes and break down fat deposits [93].

Using their hydrolytic, esterifying and acylating properties, lipases can also be used as biocatalysts of numerous cosmetic ingredients, as part of a “green chemistry” strategy. Enzymes like lipases are a part of this strategy due to their selectivity and stability and the fact that they do not usually involve the use of organic solvents for their isolation, which can be environmentally hazardous. Furthermore, enzymatic reactions reduce energy consumption, by-product formation (which is itself biodegradable) and the time required for heating, with both environmental and economic advantages and a reduction in the production costs [93].

Marine Antarctic fungi and yeasts were isolated to discover low-temperature active lipolytic enzymes. Enzymes like lipases were isolated from the phylum Basidiomycota, from sea urchin-derived *Cryptococcus laurentii* and from *Palmaria decipiens* (Rhodophyta) and *Geomyces* sp. (Myxotrichaceae) [87].

### 5.3. Invasive Species as a Source of Compounds of Interest

Climate change, a prominent concern of the 21st century, is manifested through alterations in the behavior, abundance, diversity, and distribution of marine species [94]. These changes, coupled with environmental shifts in the receiving ecosystems, create favorable conditions for the emergence of invasive alien species (IAS) or nonindigenous marine species (NIMS) [94,95]. Typically, these invasive species achieve successful invasions due to characteristics such as rapid growth rates, vegetative propagation, innovative growth strategies, high levels of sexual reproduction and broad environmental tolerance [95].

To address these species sustainably, one effective approach is to highlight their potential value in developing new products, including cosmetics [94]. Following this, we will introduce examples of invasive species possessing cosmetic appeal, which present opportunities for sustainable utilization, thereby minimizing their impact on ecosystems.

#### 5.3.1. *Sargassum* spp.

*Sargassum* spp. constitute the most abundant group of brown algae, and present a unique mechanism of photon absorption during photosynthesis and a considerable amount of carotenoids, such as fucoxanthin [96].

The largest concentration of Sargassum in the world is present in the Gulf of Mexico and the Sargasso Sea, containing mostly *S. natans* and *S. fuitans*. However, excessive amounts of *Sargassum* on beaches have been reported due to excessive blooming [96], together with the appearance of a new area of algal masses—the Great Atlantic “Sargasso” Belt—spreading across 8850 km in the Atlantic Ocean [96]. This accumulation can block sunlight penetration, originating anoxic conditions, the loss of nutrients and the production of toxic gases, with the subsequent death of species [96].

Fucoxanthin is the main pigment in *Sargassum* [96], and has skin-whitening properties due to the inhibition of tyrosinase activity and the suppression of TRP1 [10]. Furthermore, meroterpenoids found in *S. serratifolium*, which possess skin-whitening properties [16], and sargachromanol E in *S. horneri*, which activates TIMP-1 and TIMP-2 [2,16], are also some of the already-mentioned Sargassum-derived compounds.

Specifically, according to Susano et al., the extract of *S. muticum*, which occurs on several European coastlines in high amounts, brought presumptively through *Crassostrea gigas* (Mollusca) shipments, has anti-aging, anti-acne and anti-UV radiation properties, allowing the maintenance of the skin’s microbiome homeostasis [94]. Therefore, using this brown macroalgae in the cosmetic industry will not only allow a better mitigation of this spreading species, but also the retrieval of several cosmetically important active ingredients [94].

#### 5.3.2. *Ulva lactuca* and Ulvans

*Ulva lactuca* is a macroalgae from the phylum Chlorophyta. Due to its dual reproductive capacity, whether through sexual reproduction or the fragmentation of the thallus (asexual reproduction), it can rapidly spread out, covering the water surface and exhibiting an invasive pattern [97]. Therefore, *Ulva* blooms occur frequently, invading beaches and damaging marine ecosystems. These blooms have been observed worldwide, including in Europe, Asia, America and Australia; the largest *Ulva* blooms occur in Europe, on Brittany’s north coasts. As a consequence of *Ulva* biodegradation, acidic vapors can be released, leading to the death of animals [97].

Green macroalgae (with *Ulva lactuca* as an example) are rich in sulphated heteropolysaccharides, called ulvans, which contribute to the cell wall’s strength [97]. Ulvans can take part in the encapsulation of active ingredients, as shown by Selvasudha et al. [98], in whose study a combination of ulvan and sodium alginate in a 1:1 proportion resulted in the development of curcumin-loaded microbeads with a 99.2% encapsulation efficiency, significant curcumin release and a good safety and skin tolerance. Furthermore, the presence of phenolic, chlorophyll and carotenoids [97], as well as ulvans themselves [36], is also important, due to their radical scavenging activity (antioxidant) [36,97].

#### 5.3.3. *Undaria pinnatifida*

*Undaria pinnatifida* is a brown seaweed from Japan, Korea and China which has dispersed through other parts of the globe, including Europe and North and America. It is considered one of the most widespread seaweed species in the world, with an invasive behavior [94]. By outcompeting native species, *U. pinnatifida* can negatively impact the biodiversity of certain ecosystems [94]. However, this macroalgae is rich in fucoidans, a polysaccharide found in brown seaweeds, and phlorotannins, which are reported to be the most concentrated type of phenolic in *U. pinnatifida* [99].

As discussed before in Section 3, fucoidans from *U. pinnatifida*, especially LMF (low-molecular-weight fucoidans), act as “wound-healing accelerators” [37,38], promoting re-epithelization [38] and angiogenesis and reducing inflammation [6]. Furthermore, fucoidan, extracted from brown macroalgae, in which *U. pinnatifida* is included, can be used a drug carrier for controlled release systems in skin formulations [5].

### 5.4. Marine Waste Products as a Source of Compounds of Interest

Fishing, farming and processing fish and other sea products are huge generators of leftovers and waste [100], which are normally disposed of directly into the environment without any treatment [100,101]. According to the Food and Agriculture Organization of the United Nations (FAO), the annual discards from world fisheries total approximately 20 million tons, including processing leftovers, by-products and unwanted species [100]. Most of the marine discards have been seen as products with a low commercial appeal, but the utilization of this sea waste not only allows new important cosmeceutical ingredients to be discovered, but also endorses a zero-waste strategy, in line with the Sustainable Development Goals (SDGs) of the UN [100].

#### 5.4.1. Chitin and Chitosan

Chitosan is a cationic polysaccharide produced through the deacetylation of chitin, a polysaccharide found in the exoskeleton of crustaceans, through an alkalization process at high temperatures. Chitosan has medical and pharmaceutical applications, with constraints due to its molecular weight and viscosity [100]. Not only is chitosan biocompatible, biodegradable and non-toxic, but it also has antibacterial and antifungal properties [101].

Crab and shrimp shells are the most well-known sources of chitin [100]. In cosmetics, chitosan has been used as a carrier and stabilizer in sunscreen preparations, in the form of nanoparticles, and was found to have good storage and color stability in stability studies [100]. These chitosan-based nanoparticles can also be used as carriers of other cosmetic ingredients, such as anti-aging molecules [101]. Furthermore, a good cosmetic mask has been developed using a chitosan film. This mask showed good flexibility, good water retention characteristics and is compatible with other active ingredients [100].

#### 5.4.2. Collagen

Collagen is one of the most-used active ingredients in cosmetics, and it is biodegradable and biocompatible [102]. It is a structural protein found in the various connective tissues of the human body [102]. While animal-derived collagen is the most commonly selected source of collagen, religious beliefs, ethnicity, and the existence of various diseases (such as BSE or bovine spongiform encephalopathy) can pose disadvantages to its use [102]. Therefore, marine-derived collagen (and its derivatives) can be an effective and sustainable option, especially when derived from waste [100,102].

When comparing mammal-derived collagen to fish-derived collagen, there is a difference in their effectiveness on the skin. Fish collagen contains lower levels of proline and hydroxyproline, leading to reduced stability and cross-linking compatibility [102]. Moreover, the human body faces challenges in absorbing peptides with high molecular weights. Therefore, marine hydrolyzed collagen is expected to be more effectively absorbed by human skin [100]. Fish bones, skin, scales, and swim bladders are rich in a collagen matrix, especially type I collagen [102]. Collagen has the capacity to efficiently absorb water, resulting in effective moisturizing effects without causing skin irritation [100,102].

Guan et al. [103] studied the cosmeceutical properties of silver carp skin collagen (SCSC). They found that SCSC exhibited excellent foaming and emulsifying properties, along with a superior water absorption capacity and oil absorption capacity when compared to proteins sourced from terrestrial origins.

#### 5.4.3. Natural Calcium Phosphates

Calcium phosphates (CaP) occur naturally in the bones and teeth of vertebrates [100,104].

Fisheries’ byproducts, like fish bones, serve as a source of these calcium phosphates (CaPs), including hydroxyapatite, which has demonstrated effective absorption of the entire range of UV radiation [104], which is of particular interest in the formulation of broad-spectrum sunscreens [100]. Moreover, in a study conducted by Righi et al. [104], it was determined that the utilization of natural calcium phosphates offers greater environmental benefits when compared to the use of zinc oxide nanoparticles, particularly concerning issues related to eutrophication and ecotoxicity. Furthermore, these nanoparticles do not exhibit adverse effects on the four aquatic species tested (*Dunaliella tertiolecta*—Chlorophyta, *Tigriopus fulvus*, *Corophium insidiosum* and *Gammarus aequicauda*—Arthropoda) [104].

#### 5.4.4. Beach-Cast Seaweeds

Beach-cast macroalgae are naturally removed from the natural substrates derived from local drift algae or extraordinarily bloomed. The former then end up on the beach in deposits, being brought by currents, winds and tides [105].

The decomposition of excessive biomass reduces the use of the beach and drives away tourists. These biomass agglomerates also release greenhouse gases, resulting in the death of organisms due to the associated anoxic environment [105].

This biomass is easily accessible, constituting a potential source of functional ingredients to be integrated in cosmetics, with the advantage of reducing the environmental burden of this phenomenon on countries’ coasts [105]. Furthermore, parallel industries can be established in locations frequently receiving beach-casts algae, mitigating pollution and developing these regions in a sustainable way [106].

However, the discontinuous and unreliable supply of beach-cast seaweeds is a considerable challenge in terms of the use of beach-cast macroalgae [105].

## 6. Conclusions

Years of research and development have underscored the vast potential of marine-derived ingredients in the cosmetic industry. This potential is attributed to the multitude of beneficial biological properties inherent in these products and their eco-friendly, sustainable nature. In the present day, there is a growing trend towards sustainable cosmetics and natural ingredients, driven by mounting concerns regarding the environmental and human health impacts of cosmetics. In this review, we have not only highlighted the benefits and positive aspects of incorporating marine-derived ingredients, but also delved into the challenges and implications associated with their utilization. Anti-aging, anti-tyrosinase, anti-inflammation, and wound healing—these are some of the properties we can find in marine species, ranging from the well-known algae to the less-known sea cucumbers, without forgetting fish and crustaceans. Looking ahead, it is essential to enhance the extraction methods. This is imperative not only to mitigate the potential risks of biological and chemical contamination, which can be addressed through various conventional and advanced techniques, but also to address the relatively high sustainability burden stemming from the substantial volume of raw material required to obtain these compounds and also from the use of hazardous solvents in the extraction phases. This review also delved into the existing regulatory framework governing marine-derived cosmetics. Regulation 1223/2009/EC [72], coupled with ISO guidelines and CEN technical reports, constitute the primary regulatory documents concerning the incorporation of marine-derived ingredients into cosmetics. With the growing adoption of these ingredients, there is a pressing need for an update to the regulatory and normative framework in the coming years. Another challenge pertains to the prevalence of iodine in most marine species, given that it is among the substances prohibited in cosmetic products.

Finally, solutions rooted in “blue biotechnology” hold the potential to offer more sustainable alternatives for cosmetics. One of the primary advantages of these scientific advancements is their reduced environmental and societal impact. Exploring invasive species to mitigate their adverse effects and recycling oceanic waste and byproducts represent innovative approaches to minimize the environmental footprint of cosmetics while delivering novel and cutting-edge products to consumers. Furthermore, a deeper comprehension of sustainability principles applied throughout the cosmetic product lifecycle is essential, particularly to minimize the associated carbon footprint and greenhouse gas emissions. The utilization of marine-derived cosmetic ingredients is not a concept we envision solely for the future; it is already a reality in the present. To address the challenges associated with this domain, the cosmetic industry must rely on innovation, creative thinking, and technology, with a particular focus on incorporating “blue biotechnology” solutions.

## Figures and Tables

**Table 1:** Marine-derived polysaccharides and their cosmetic properties (Source: COSiNG website).

Polysaccharides	Cosmetic Properties to CosIng Database	Species Where These Polysaccharides Are Found (e.g.,)
Alginates and their salts (calcium, sodium, magnesium, ammonium and potassium)	Binding Emulsion stabilizing Film-forming Humectant Viscosity-controlling	Brown macroalgae (e.g., *Ascophyllum nodosum, Laminaria hyperborea, Laminaria digitata*) [4]
Carrageenans (including hydrolyzed carrageenan)	Binding Emulsion-stabilizing Film-forming Skin-conditioning Viscosity-controlling	*Kappaphycus* and *Eucheuma* genera [4]
Agar and agarose	Binding Fragrance Viscosity-controlling Skin conditioning	Red macroalgae (*Gelidium* and *Gracilaria* species) [4]
Fucoidan	Skin-conditioning Skin protector	Brown macroalgae (e.g., *Sargassum stenophyllum*, *Fucus vesiculosus*) [4]
Xylans	Film-forming Skin-conditioning	Green macroalgae (Bryopsidales order) [4]
Mannans	Film-forming Skin-conditioning	Green macroalgae (Bryopsidales order) [49]

## Data Availability

The original data presented in the study are included in the article/Supplementary Materials; further inquiries can be directed to the corresponding author.

## Supplementary Materials

The following supporting information can be downloaded at: https://www.mdpi.com/article/10.3390/md21120620/s1.
