# Supplementary material for: Exploring the Potential of Using Marine-Derived Ingredients: From the Extraction to Cutting-Edge Cosmetics
Source: Mar Drugs. 2023 Nov 29;21(12):620. doi: 10.3390/md21120620 (PMC10744737; doi:10.3390/md21120620)
Supplement: Supplementary file 1 [file marinedrugs-21-00620-s001.zip › marinedrugs-2704730-supplementary-XML.pdf]

marine drugs

Review

Exploring the potential of using marine-derived ingredients: from the extraction to cutting-edge cosmetics

João Pedro Costa [1], Luísa Custódio[2] and Catarina Pinto Reis [3, 4*]

[1] Faculty of Pharmacy, Universidade de Lisboa, Av. Professor Gama Pinto, 1649-003 Lisboa, Portugal;

[2] Centre of Marine Sciences, University of Algarve, Faculty of Sciences and Technology, Ed. 7, Campus of Gambelas, 8005-139 Faro, Portugal;

[3] Research Institute for Medicines, iMed.ULisboa - Faculty of Pharmacy, Universidade de Lisboa, Av. Professor Gama Pinto, 1649-003 Lisboa, Portugal;

[4] Instituto de Biofísica e Engenharia Biomédica (IBEB), Faculdade de Ciências, Universidade de Lisboa, Campo Grande 1749-016 Lisboa, Portugal.

Citation: Costa, J.P.; Custódio, L.; Reis, C.P. Exploring the potential of using marine-derived ingredients: from the extraction to cutting-edge cosmetics. *Mar. Drugs* **2023**, *21*, 620. https://doi.org/10.3390/md21120620

Academic Editor: Leonel Pereira and Rui Pedro Gonçalves Pereira

Received: 23 October 2023
Revised: 21 November 2023
Accepted: 21 November 2023
Published: 4 December 2023

Copyright: © 2023 by the authors. Licensee MDPI, Basel, Switzerland. This article is an open access article distributed under the terms and conditions of the Creative Commons Attribution (CC BY) license (https://creativecommons.org/licenses/by/4.0/).

1. Conventional methods of extraction

Conventional methods of extraction include infusion, percolation, Soxhlet extraction, maceration, steam distillation, among others [54–63]. These constitute less environmentally friendly processes, requiring many hours of extraction and, in some cases, large amounts of chemical solvents and raw material [54].

Sometimes, due to the difficult penetration of the solvents inside cells, cell disruption methods are therefore used to improve the efficiency of extraction. Physical processes, such as grinding with a homogeniser and promoting osmotic stress, have been reported to improve the extraction efficiency of a number of active ingredients, especially algal proteins [54].

The choice of solvent depends on the solubility of the target substance [58], impacting selectivity and allowing for a high efficiency in the extraction process – for instance, phycocolloids are extracted in hot water due to its water solubility [54]. The heating process usually involves convection through the solvent and conduction from the surfaces to the nucleus of the matrix particles [57]. Other properties influence the yield of extraction, such as the diffusion coefficient, solvent viscosity, rate of mass transfer of the extracted substance and conditions under which the extraction is conducted – time, temperature and pressure [58].

1.1. Solid-liquid extraction (SLE)

SLE includes different processes, such as maceration, infusion and percolation, among others [59-60]. It is the most common extraction method for antioxidant material and has been successfully used to extract polyphenolic compounds [57]. Different variables are evaluated, including biomass-solvent ratio, percentage of solvent, time and temperature of incubation [60]. The solvents utilized in the different maceration processes are usually organic [59] and include methanol (MeOH) and ethanol (EtOH) at different percentages, with ethanol as the preferred solvent [60].

Using a suitable liquid phase, this technique allows the isolation of desired soluble constituents from a solid or semisolid matrix, during which the solute migrates from the solid matrix into the solvent. Removing the excess water and grounding samples into powder are essential to improve the process yield, as it will, respectively, increase recovery of non-polar compounds and increase their surface area [59].

Therefore, SLE is a high-energy technique, besides being very simple and relatively low cost [59].

3.1.2. Soxhlet-assisted extraction (SAE)

Soxhlet assisted extraction (SAE) is extensively used, especially for the extraction of molecules with limited solubility in a solvent and in the presence of insoluble impurities. Through the continuous passage of the solvent through the sample matrix, using boiling at temperatures below the solvent's boiling point and condensation, the compounds of interest are transferred from the solid to the solvent [59,60].

The polarity of the solvent, as well as its capacity to dissolve the desired analytes, are critical aspects that affect the efficiency of extraction – the most used are organic liquids, such as ethanol, methanol, acetone and hexane [59]. SAE is widely used for the recovery of lipids and phenolic antioxidants, such as carotenoids, from seaweeds [58,59].

3.2. Alternative methods of extraction

Advanced, alternative or contemporary extraction methods include supercritical fluid extraction (SFE), microwave-assisted extraction (MAE), ultrasound-assisted extraction (UAE), enzyme-assisted extraction (EAE) and electro-technologies [2,57,59].

These methods of extraction are developed under a "green chemistry" approach, with a reduction of labor and energy needed, with safer, more environmentally-friendly solvents and less production of waste and gas emissions [58].

3.2.1. Microwave-assisted extraction (MAE)

MAE is the most cost-effective used technology at the laboratory scale nowadays [57] and a preferred method for extracting ulvan and rhamnan sulfate due to the possibility of eliminating toxic solvents [2]. This technique uses microwave irradiation (nonionizing waves with a frequency between 300 MHz and 300 GHz), generating heat in a few seconds through collisions and frictions between molecules [57] and is environmentally friendly and economically appealing because of its reduced extraction time and solvent consumption [2]. MAE can be operated in open and closed vessels, with the first being safer, effective and able to process larger samples [2]. In this method, pressure and temperature are key factors and offer some selectivity to the extraction process. When high temperatures and pressures are applied, the yield of the phenolic compounds seems to be reduced; on the other hand, a lower yield of polysaccharides is obtained using low pressure for a longer period of time [2].

Fucoidan, fucoxanthin, phenols and polysaccharides are some examples of compounds that have already been extracted from marine products using MAE [61].

3.2.2. Ultrasound-assisted extraction (UAE)

UAE uses high-frequency sound waves (> 20 KHz), which create bubbles and zones of high and low pressure [1,4]. The passage of these waves induces cavitation in the solvent and the formation of cavitation bubbles, which increase the surface area between solid and liquid phases and the mass transfer from the biological matrix [4,54,57]. The violent implosion of these cavitation bubbles also facilitates algal cell wall breakdown and enhances extraction efficiency [54,57], through the creation of microscopic regions of extreme pressure and temperature [54].

This cost-effective and efficient technique is usually used for the extraction of phenolic compounds [2] and can be used as a pretreatment to SLE by destroying the biomaterial and making the target compounds more accessible [59,60], using ultrasonic bath (indirect sonication) or an ultrasonic probe (direct sonification) [59].

Pressure, temperature, intensity and frequency of the waves, surface tension and viscosity of the solvent are parameters that influence the extraction process [57], with higher temperature increasing the yield [2].

3.2.3. Enzyme-assisted extraction (EAE)

EAE is an eco-friendly approach that uses enzymes such as peptidases, glycosidases and carbohydrases to cause a disruption on the algae cell wall, allowing for a release of the intracellular metabolites [2,57]. This method is analogous to the previously mentioned method of physical disruption of the cell wall [2].

Some advantages of this method include the conversion of water-insoluble materials into water-soluble materials, the preservation of the original efficacy of the compounds, a good catalytic efficiency, a high specificity and a significant process scalability [2,57]. It can also be considered the greenest among all the methods, as it does not use any harmful chemicals or organic solvents [57].

On the other hand, this method shows some limitations, including the cost of enzymes, some of these with prohibitive costs, the lack of enzymes and the difficulty in maintaining bioreactor conditions [2,57]. These limitations can be overcome by the use of other extraction methods coupled with EAE, such as ultrasound assisted enzymatic extraction (UAEE) or enzyme assisted high pressure extraction [2,62].

3.2.4. Supercritical fluid extraction (SFE)

SFE is a technology that uses carbon dioxide and water as supercritical solvents [57]. SFE operates in the supercritical state of the solvent, above the critical pressure and temperature of the fluid, in which the supercritical fluid density is similar to a liquid and the viscosity is low in a range between liquid and gas, so that this can rapidly penetrate into solid materials and improve the extraction process yield. These conditions promote the transfer of solute from the matrix to the solvent [56,57,59].

Supercritical carbon dioxide (SC-CO2) is the most used solvent because it is non-toxic, non-inflammable, non-explosive, safe and environmentally available [57,59]. Other possible supercritical solvents, like methanol or propylene, do not meet the criteria of "green chemistry" [57]. However, due to its low polarity, SC-CO2 is only applied to recover non-polar or mid-polar compounds, such as fatty acids, carotenoids and essential oils [2,59]. More polar co-solvents, such as ethanol, can be used together with SC-CO2 to expand the range of compounds achievable by this technology, with low toxicity, but with possible decreased selectivity [59].

Pressure and temperature affect the selectivity and solubility of the various compounds in the supercritical fluid – carbon dioxide has a relatively low critical temperature (31.1ºC) and pressure (73.8 bar), making it an excellent solvent for sensible compounds [59]. In general, increasing pressure and temperature results in an increased extraction rate, due to an easier dissipation of the molecules through the matrices and a lower surface tension of SC-CO2 [59]. Other factors, such as water content, particle size and extraction time, are also involved [59].

Overall, SFE is considered advantageous, but also expensive due to high equipment costs and energy consumption, making the scale-up process yet unfeasible [57]. Some of the already mentioned applications for SFE include the extraction of astaxanthin, fatty acids, fucoxanthin and lipids [61].

3.2.5. Pressurized liquid extraction (PLE)

PLE is based on pressurized solvents under values of temperature and pressure ranging between 50-300ºC and 35-200 bar, respectively [57,59]. The solvent is heated above its normal pressure and temperature boiling point, but below its critical point, resulting in a decreased solvent viscosity and surface tension and a better penetration of the matrix, with an improved mass transfer to the solvent and extraction rate [57,59].

Temperature influences the extraction's efficiency and selectivity greatly, with higher temperatures being linked to better extraction yields. Nevertheless, this technique cannot be used for thermolabile compounds [59].

When water is used, this technique is also called subcritical water extraction (SWE). Water is the most used solvent, as it does not generate toxic waste. As water's affinity towards less polar compounds is increased with temperature, the combined high temperature and pressure allows for an increased desorption of target compounds from the material, overall increasing the extraction kinetics [59].

Therefore, PLE allows for a high extraction efficiency, less solvent consumption and a shorter extraction time, with phenolic compounds, polysaccharides and aminoacids as some examples of extracted compounds [57,61].

3.2.6. Electro-technologies

Electro-technologies include pulsed electric field (PEF), ohmic heating (OH), moderate electric field (MEF) and high-voltage electric discharges (HVED) in which the synergistic effects of temperature and electric fields are applied [57,63].

PEF has been used as a cell disruption technique in algae [54], as it enhances cell membranes' permeability by inducing reversible or irreversible electroporation [54,57], through the application of very short pulses of very high voltage electric fields [57]. Electroporation enables the introduction of foreign components to cells, including DNA, proteins and drugs [54]. PEF is extremely versatile, efficient, requires low energy and water consumption, does not require chemicals, and generates low heat. PEF is being used to induce stress and thus extract compounds more easily [57]. Numerous studies have shown PEF's capacity of potentially increasing the yield of extraction of several compounds, including lipids, carbohydrates, carotenoids and chlorophyll [54].

In OH a moderate electric field is applied to heat a sample. A homogeneous and instantaneous transmission of thermal energy and the fast heating enhances cell membrane permeability, making it possible to recover high-value molecules [54]. Besides the thermal effects, electroporation of cellular tissues, with membrane damage and solutes diffusion, has also been reported, thus facilitating the extraction of bioactive compounds [54].

MEF exposes the sample matrix to low electric fields (between 1 and 1000 V/cm), where electric frequencies in the range of Hz up to tens of kHz are applied. High-voltage electric discharges or HVED typically apply 40-60 kV/cm for 2–5 μs electrical property [63].

References

2. Thiyagarasaiyar, K., Goh, B. H., Jeon, Y. J., & Yow, Y. Y. (2020). Algae metabolites in cosmeceutical: An overview of current applications and challenges. In Marine Drugs (Vol. 18, Issue 6). MDPI AG. https://doi.org/10.3390/md18060323
54. Bleakley, S., & Hayes, M. (2017). Algal proteins: Extraction, application, and challenges concerning production. Foods, 6(5), 1–34. https://doi.org/10.3390/foods6050033
56. Chojnacka, K., & Kim, S.-K. (2015). Introduction of Marine Algae Extracts. https://doi.org/10.1002/9783527679577.ch1
57. Matos, G. S., Pereira, S. G., Genisheva, Z. A., Gomes, A. M., Teixeira, J. A., Rocha, C. M. R., Alexandre, C., Manuel, J., Saraiva, A., & Pintado, M. (2021). foods Advances in Extraction Methods to Recover Added-Value Compounds from Seaweeds: Sustainability and Functionality. https://doi.org/10.3390/foods
58. Łęska, B., Messyasz, B., & Schroeder, G. (2018). Application of Algae Biomass and Algae Extracts in Cosmetic Formulations. In Algae Biomass: Characteristics and Applications (pp. 89–101). Springer International Publishing. https://doi.org/10.1007/978-3-319-74703-3_8
59. Quitério, E., Grosso, C., Ferraz, R., Delerue-Matos, C., & Soares, C. (2022). A Critical Comparison of the Advanced Extraction Techniques Applied to Obtain Health-Promoting Compounds from Seaweeds. In Marine Drugs (Vol. 20, Issue 11). MDPI. https://doi.org/10.3390/md20110677
60. Lourenço-Lopes, C., Garcia-Oliveira, P., Carpena, M., Fraga-Corral, M., Jimenez-Lopez, C., Pereira, A. G., Prieto, M. A., & Simal-Gandara, J. (2020). Scientific approaches on extraction, purification and stability for the commercialization of fucoxanthin recovered from brown algae. In Foods (Vol. 9, Issue 8). MDPI AG. https://doi.org/10.3390/foods9081113
61. Michalak, I., & Chojnacka, K. (2014). Algal extracts: Technology and advances. Engineering in Life Sciences, 14(6), 581–591. https://doi.org/10.1002/elsc.201400139
62. Nadar, S. S., Rao, P., & Rathod, V. K. (2018). Enzyme assisted extraction of biomolecules as an approach to novel extraction technology: A review. Food Research International, 108, 309–330. https://doi.org/10.1016/j.foodres.2018.03.006
63. Gupta, A. K., Seth, K., Maheshwari, K., Baroliya, P. K., Meena, M., Kumar, A., Vinayak, V., & Harish. (2021). Biosynthesis and extraction of high-value carotenoid from algae. In Frontiers in Bioscience – Landmark (Vol. 26, Issue 6, pp. 171–190). Frontiers in Bioscience. https://doi.org/10.52586/4932

Disclaimer/Publisher's Note: The statements, opinions and data contained in all publications are solely those of the individual author(s) and contributor(s) and not of MDPI and/or the editor(s). MDPI and/or the editor(s) disclaim responsibility for any injury to people or property resulting from any ideas, methods, instructions or products referred to in the content.
